# Direct Cardiac Reprogramming: Progress and Promise

**DOI:** 10.1155/2018/1435746

**Published:** 2018-03-13

**Authors:** James L. Engel, Reza Ardehali

**Affiliations:** ^1^Molecular, Cellular and Integrative Physiology Graduate Program, University of California, Los Angeles, CA 90095, USA; ^2^Division of Cardiology, Department of Internal Medicine, David Geffen School of Medicine, University of California, Los Angeles, CA 90095, USA; ^3^Eli and Edythe Broad Stem Cell Research Center, University of California, Los Angeles, CA 90095, USA

## Abstract

The human adult heart lacks a robust endogenous repair mechanism to fully restore cardiac function after insult; thus, the ability to regenerate and repair the injured myocardium remains a top priority in treating heart failure. The ability to efficiently generate a large number of functioning cardiomyocytes capable of functional integration within the injured heart has been difficult. However, the ability to directly convert fibroblasts into cardiomyocyte-like cells both *in vitro* and *in vivo* offers great promise in overcoming this problem. In this review, we describe the insights and progress that have been gained from the investigation of direct cardiac reprogramming. We focus on the use of key transcription factors and cardiogenic genes as well as on the use of other biological molecules such as small molecules, cytokines, noncoding RNAs, and epigenetic modifiers to improve the efficiency of cardiac reprogramming. Finally, we discuss the development of safer reprogramming approaches for future clinical application.

## 1. Introduction

Heart failure (HF), the leading cause of death and hospitalizations worldwide, results from a myriad of cardiovascular diseases that lead to the death or dysfunction of cardiomyocytes. With a prevalence of 38 million people worldwide, it places a significant financial burden on health care systems, with an estimated $30 billion of annual spending in just the United States alone [1, 2]. Despite recent advances in the care and management of HF patients, the prognosis of advanced HF remains dismal at 50% survival at 5 years, a rate often lower than that of many cancers [3, 4]. Considering that the pathophysiology of HF involves death or dysfunction of the cardiac myocyte, new therapeutic strategies for heart regeneration may offer hope to this intractable disease.

The human adult heart lacks endogenous repair mechanisms to fully restore cardiac function after an insult; thus, the ability to regenerate and repair the injured myocardium remains a top priority in treating HF. However, the ability to efficiently generate a large number of functioning cardiomyocytes capable of functional integration within the injured heart has remained an obstacle. Current cell therapies are focused on three main approaches: (1) induction of endogenous cardiomyocytes to undergo proliferation and repopulate the damaged myocardium, (2) transplantation of cardiovascular progenitor cells (CPCs) or cardiomyocytes generated through the differentiation of pluripotent stem cells, and (3) direct reprogramming of somatic cells to cardiomyocytes or expandable CPCs without transitioning through a pluripotent intermediate. This review is focused on the last approach. Direct reprogramming was first reported in 1987 when a single cDNA encoding MyoD was transfected into fibroblasts converting them into muscle myoblasts [5]. A few years later, MyoD was identified as the master regulator gene for skeletal muscle development [6]. The ability to directly reprogram adult cells to a desirable fate demonstrates an immense potential of this powerful tool for tissue regeneration and replacement. Since the identification of MyoD, there has been extensive focus on the identification of master regulator(s) for other cell lineages and this search has led to the successful conversion of mature cells into other cells types including myoblasts, neurons, hepatocytes, intestinal cells, blood progenitor cells, and cardiomyocytes [5, 7–11].

In this review, we describe the insights and progress that have been gained from the investigation of direct cardiac reprogramming, with a focus on the use of key transcription factors and other cardiogenic genes. Furthermore, we discuss the use of other biologics and small molecules to improve the efficiency of cardiac reprogramming and the development of safe reprogramming approaches for clinical application.

## 2. Reprogramming of Somatic Cells to Cardiomyocyte-Like Cells by Overexpression of Key Cardiac Transcription Factors

Direct reprogramming of fibroblasts into cardiomyocyte-like cells was first reported in 2010 using viral overexpression of three important cardiac developmental transcription factors (TFs), *Gata4*, *Mef2c*, and *Tbx5* (GMT) in mouse cardiac and tail-tip fibroblasts [11]. Ieda et al. used an iterative screening approach in which 14 factors were removed one by one to identify those that were dispensable for direct reprogramming. This process ultimately identified GMT as the factors sufficient to induce conversion of fibroblasts to cardiomyocyte-like cells without transitioning through a progenitor state. TBX5 is an important T-box TF involved in early cardiac development that directs formation of the primary heart field through a coordinated but yet complex interaction with other TFs [12]. One such interaction is with GATA4, a member of the GATA family zinc-finger TFs, which modifies the chromatin structure allowing other TFs such as NKX2–5 to bind to their targets and fully activate the cardiac transcriptional program [13]. MEF2c, a MADS box transcription enhancer factor, is important for the formation of the secondary heart field through its interaction with other cardiac TFs [14]. After the establishment of GMT as the core TFs for direct cardiac reprograming, much of the focus transitioned to improving the reprogramming efficiency and/or the function of the induced cardiomyocyte-like cells (iCMs) through addition of other important cardiac TFs to GMT. This was mainly due to the poor efficiency of reprogramming, reported to be 4.8% cardiac troponin T+ (cTnT+) cells in the original paper. Additionally, it was soon noted that GMT alone was insufficient to convert human fibroblasts to iCMs.

One of the first TFs added to GMT was the bHLH TF HAND2 (referred to as GMHT). In cardiac development, HAND2 plays an important role in the formation of the ventricular chambers through interaction with GATA4 and NKX2–5 [15]. GMHT treatment of mouse embryonic fibroblasts (MEFs) resulted in iCMs expressing low levels of sarcomeric proteins and displayed immature characteristics of the main cardiac cell types (atrial, ventricular, and pacemaker) [16]. In an effort to increase transcriptional activity of GMHT, the transactivation domain of MyoD was fused to each G, M, H, or T and overexpressed in mouse fibroblasts. When *MyoD* was fused to *Mef2c*, a 15-fold increase in reprogramming efficiency was observed [17]. Other TFs that are essential during cardiovascular development have also been studied for direct reprogramming. NKX2–5, a homeobox TF critical for normal heart morphogenesis, was overexpressed in mouse fibroblasts in addition to GMHT. This combination resulted in a more than 50-fold increase in the efficiency of cardiac reprogramming compared to GMT alone and produced iCMs with mature cardiomyocyte marker expression, robust calcium oscillation, and spontaneous beating [18].

Additionally, an alternative screening approach that surveyed triplet combinations of 10 important cardiac TFs revealed that *Tbx5*, *Mef2c*, and *Myocd*, a developmental regulator of cardiomyocytes and smooth muscle cells, were able to induce a more complete cardiac phenotype than GMT in mouse fibroblasts [19]. Likewise, a combinatorial screen of 10 transcription factors added to GMT in MEFs identified a combination of cocktails that resulted in successful reprogramming. GMT plus *Myocd* and *Srf*, a TF important in mesoderm formation, or GMT plus *Myocd*, *Srf*, *Mesp1*, another mesoderm-inducing TF, and *Smarcd3*, a chromatin structure-altering protein, enhanced reprogramming and the expression of cardiac sarcomeric proteins over GMT alone [20].

Despite the successes of TF overexpression to reprogram murine cells, similar approaches to reprogram human somatic cells have been more difficult to achieve. Only a few studies have reported successful reprogramming of human cells to iCMs using TFs alone. The first of these studies reported a combination of the E26 transformation-specific (ETS) TF family member *ETS2* and *MESP1* proteins to induce reprogramming of human dermal fibroblasts to cardiac progenitors [21]. Another study using GMT with *MESP1* and *MYOCD* in human cardiac and dermal fibroblasts was sufficient to induce expression of multiple cardiac-specific proteins, increase a broad range of cardiac genes, and exhibit spontaneous calcium transients [22]. The third report showed that expressing GMT along with *ESSRG* (a transcriptional activator), *MESP1*, *MYOCD*, and *ZFPM2* (a modulator of GATA proteins) in human fetal cardiac fibroblasts and neonatal skin fibroblasts enhanced cardiac reprogramming, sarcomere formation, calcium transients, and action potentials [23]. Results of TF-based reprogramming are summarized in Table 1.

## 3. Improving the Efficiency of Direct Reprogramming with Biological Molecules

Despite the successes of direct reprogramming using forced expression of cardiac TFs, the efficiency remains low. The initial report on direct conversion of fibroblasts to cardiomyocyte-like cells noted an efficiency of 4.8%. In an effort to improve reprogramming efficiency, many methods have been developed using additional molecules. These additives can be classified into three major categories: inhibitors/cytokines, noncoding RNAs, and epigenetic modifiers. A summary of these reprogramming experiments is presented in Table 2.

### 3.1. Inhibitors/Cytokines

**Figure 1:**
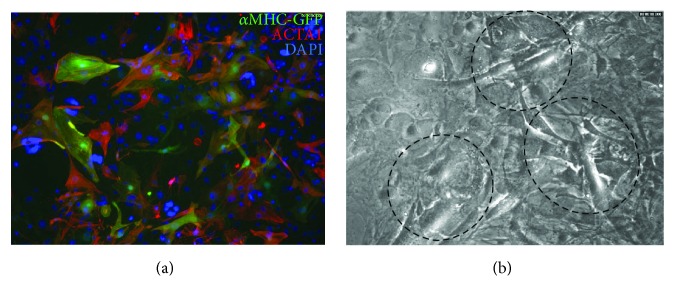
GMT + A83-01 reprogramming in MEFs. (a) Immunocytochemistry for the cardiac markers *α*MHC-GFP and *α*-actinin (ACTA1). (b) Video snapshot showing beating areas of reprogrammed cells (outlined areas).

A potential approach to improving reprogramming is to inhibit the endogenous signaling pathways and gene programs that maintain the distinct properties of fibroblasts. One of the major signaling pathways active in fibroblasts is the transforming growth factor- (TGF-) *β* pathway. TGF-*β* has diverse and pleotropic effects through its activation and signaling. The downstream effect of the TGF-*β* signaling pathway involves phosphorylation of receptor-regulated SMADs that ultimately activate TFs that participate in the regulation of target gene expressions, many of which are critical in fibroblast activation and proliferation. Since inhibition of TGF-*β* has been shown to increase mouse embryonic stem cell differentiation to cardiomyocytes [24, 25], it was hypothesized that TGF-*β* inhibition could improve reprogramming. The TGF-*β* inhibitors SB431542 and A83-01 have been added to various reprogramming combinations and have shown an increase in reprogramming efficiency. SB431542 is a selective and potent inhibitor of the TGF-*β* pathway through suppression of the activin A receptors ALK5, ALK4, and ALK7. A83-01 is also a selective inhibitor of ALK5, ALK4, and ALK7 but is more potent than SB431542 in its inhibition and effectively blocks phosphorylation of Smad2. When SB431542 was combined with GMHT, a 5-fold increase in reprogramming efficiency was observed in both MEFs and mouse adult cardiac fibroblasts [26]. Likewise, we observed an increase in reprogramming efficiency, with frequent areas of spontaneous contraction and enhanced expression of cardiac contractile proteins when we reprogrammed MEFs with GMT and A83-01 cells (Figure 1). Furthermore, when GMT and SB431542 were combined with WNT inhibition by XAV939, reprogramming efficiency was increased 8-fold in cardiac fibroblasts with respect to GMT alone [27]. In addition to the TGF-*β* pathway, other profibrotic and intracellular signaling pathways such as the Rho-associated kinase, JAK/STAT, Notch and Akt pathways have been targeted to improve reprogramming [28–31].

The utility of other molecules to enhance cardiac reprogramming has been inspired by using cytokines and/or modulators that are critical in mammalian cardiac development, many of which are commonly used in the differentiation of cardiomyocytes from pluripotent stem cells. Fibroblast growth factor-2 (FGF2), FGF10, and vascular endothelial growth factor (VEGF) in combination with GMT or GMHT in MEFs and mouse tail-tip fibroblasts showed an increase in the number of iCMs that spontaneously contract [32]. These approaches also accelerated maturation of iCMs *in vitro*, and thus, the activation of the important developmental pathway during reprogramming warrants further research.

### 3.2. Noncoding RNAs

MicroRNAs (miRNAs) are small noncoding RNAs that induce degradation or inhibit translation of target mRNAs. miRNAs are an attractive additive to reprogramming since they play important roles in the posttranscriptional regulation of cardiac gene expression and have critical function in almost every stage of heart development. miRNA-1, miRNA-133, miRNA-208, and miRNA-499 have been shown to be cardiac- and muscle-specific and play important roles in cardiac development and function. miRNA-1, contributing to ~40% of total miRNAs in the heart, has been shown to promote cardiomyocyte proliferation and suppress apoptosis during development; however, its function in cardiac reprogramming remains unknown [33]. miRNA-133 is important in orchestrating cardiac development, gene expression, growth, and function [34]. It also promotes cardiomyocyte proliferation by repressing the transcriptional regulator SNAI1 and silences fibroblast gene signatures during reprogramming [35]. These two miRNAs have been used in combination with other factors to successfully enhance cardiac reprogramming. A novel approach was reported recently where a combination of miRNAs promoted direct conversion of cardiac fibroblasts into cardiomyocyte-like cells without the need for forced expression of exogenous TFs. A combination of miRNA-1, miRNA-133, miRNA-208, and miRNA-499 was reported to be sufficient to convert mouse cardiac fibroblasts into iCMs without the addition of other factors *in vivo* [36]. The potential mechanism for this effect is thought to be due to altered H3K27 methyltransferase and demethylase expression, which leads to changes in the epigenetic landscape of fibroblasts to induce their conversion into cardiomyocyte-like cells. A 10-fold increase in miRNA-mediated murine cardiac fibroblast reprogramming was observed when miRNA-1, miRNA-133, miRNA-208, and miRNA-499 were combined with JAK inhibitor I [30].

Similarly, when miRNA-133 was used in conjunction with GMT, *Mesp1*, and *Myocd* or GHT, *Myocd*, and miRNA-1, the reprogramming efficiency was increased in both human and mouse fibroblasts by repressing Snai1 and silencing fibroblast gene signatures [35, 37]. Zhao et al. used a combination of GMHT, miRNA-1, miRNA-133, miRNA-208, miRNA-499, Y-27632, and A83-01 in MEFs and mouse adult fibroblasts to achieve ~60% cardiac troponin T+ and 60% *α*-actinin+ iCMs [29]. miRNA-590, a miRNA that can induce adult cardiomyocyte proliferation, was recently shown to be able to replace *HAND2* and *MYOCD* in GMT direct reprogramming experiments using human and porcine fibroblasts [38, 39]. While GMT was initially shown to be sufficient for cardiac reprogramming, further studies have indicated that a multiprong approach may be necessary to enhance reprogramming and could hold great promise for future *in vivo* clinical application.

Long noncoding RNAs (lncRNAs) are a heterogeneous group of transcripts longer than 200 nucleotides that exert major regulatory roles in gene expression during development and disease through many different mechanisms. Recent advances in sequencing and analysis technologies have allowed many lncRNAs to be identified, but due to their complex and multiple mechanisms of action as well as to the low interspecies conservation, it has been difficult to decipher biological functions of many lncRNAs [40]. A list of cardiac lncRNAs that are involved in cardiac differentiation, development, and contractile function has been reviewed [41]. Braveheart (*Bvht*) and *Fendrr* play a critical role in cardiac lineage commitment by regulating the transition from mesoderm to CPCs through activation of key cardiac development genes and TFs including some of those studied above [42–44]. *Hotair*, *Chaer*, and other lncRNAs have also been shown to regulate the epigenetic landscape in cardiac development by regulating proteins involved in histone modification at targeted promoters [45, 46]. lncRNAs play an extensive role in the regulation of cardiac development and gene expression; therefore, it may be advantageous to explore the use of lncRNAs in direct reprogramming studies; however, no direct reprogramming studies have been published using lncRNAs.

### 3.3. Epigenetic Modifiers

Reprogramming of one somatic cell type to another requires the activation and repression of multiple sets of genes, leading to vast genomic changes. The epigenetic landscape plays an important role in determining the reprogramming efficiency as accessibility of TFs to their DNA targets is critical. During reprogramming, epigenetic marks such as histone methylation, acetylation, and ubiquitination must be added and removed from fibroblast- and cardiac-specific genes. These modifications will suppress expression of fibroblast genes while activating cardiac genes by remodeling chromatin structure to allow or restrict the access of TFs to their target genes. It has been shown that during cardiac direct reprogramming, the trimethylated histone H3 of lysine 27 (H3K27me3), which marks inactive chromatin, increases at fibroblast promoters and decreases at cardiac promoters while the activated chromatin mark H3K4me3 shows the opposite pattern at important loci [11, 47]. Moreover, the activating H3K4me2 histone mark has been shown to be increased at the regulatory regions of miRNA-1 and miRNA-133 [29]. To this end, attempts to improve direct cardiac reprogramming have been carried out using modulators of epigenetic marks. Bmi1 was identified as a barrier to reprogramming by modifying histone marks at key cardiogenic loci, thus inhibiting iCM induction. When *Bmi1* activity was knocked down, the active histone mark, H3K4me3, was increased while the repressive H2AK119ub mark was reduced, leading to increased cardiac gene expression at important loci [48]. In nonintegrative and *in vivo* reprogramming experiments discussed later in this review, other epigenetic modifiers that inhibit histone methyl transferases and histone demethylase have been used. The importance of epigenetic landscape and changes that happen during reprogramming have recently begun to be unraveled using a single-cell transcriptomic approach by Liu et al. [49]. These results highlight the complexity of the reprogramming process and the importance of the influence of a variety of factors, warranting additional research into the sequential addition of TFs, noncoding RNAs, cytokines, inhibitors, and epigenetic modifiers to further improve the reprogramming efficiency.

## 4. Direct Reprogramming to Expandable Cardiac Progenitor Cells

Another recent approach of clinical promise is the generation of expandable CPCs by direct reprogramming. The goal of this approach is to safely create CPCs *in vitro* that can then be expanded in culture before transplantation into the injured heart. Upon transplantation, the CPCs will differentiate into three major cells of the heart; cardiomyocytes, endothelial cells, and smooth muscle cells. Two groups were able to successfully generate expandable CPCs using unique reprogramming cocktails containing a variety of biomolecules described above. Lalit et al. generated CPCs from fibroblasts using Mesp1, Tbx5, Gata4, Nkx2–5, and Baf60c, a chromatin remodeling protein. *In vitro* expansion and maintenance of a CPC state were achieved using a Wnt activator, BIO, and a JAK/STAT activator, LIF [50, 51]. On the other hand, Zhang et al. used a chemical approach to reprogramming fibroblasts to CPCs. Generation, expansion, and maintenance of CPCs were achieved by the addition of BMP4, activin A, CHIR99021, and SU504 (a FGF, VEGF, and PDGF signaling inhibitor) [52]. Both groups were able to show that their reprogrammed CPCs maintained their characteristics for many passages in culture and could generate cardiomyocytes, endothelial cells, and smooth muscle cells both *in vitro* and *in vivo* when transplanted. Direct reprogramming of fibroblasts to CPCs represents a scalable method for the generation of multiple cardiac cell types for clinical applications; however, this approach has not yet been applied to human cells.

## 5. Progress of *In Vivo* Direct Reprogramming

The ultimate goal of direct reprogramming is to be able to repair the damaged myocardium after injury. Direct reprogramming offers two potential approaches for heart regeneration: (1) transplantation of reprogrammed fibroblasts into the infarcted heart and (2) reprogramming resident cardiac fibroblasts directly to cardiomyocytes. The first attempt at cardiac regeneration using direct reprogramming was carried out using cardiac fibroblasts that were transduced with GMT for 1 day and then transplanted into mouse hearts [11]. Analysis of these cells posttransplantation revealed that they successfully generated cardiomyocyte-like cells *in vivo*. Other studies have used *in vivo* transplantation of reprogrammed cells to test their regenerative potential. However, cell transplantation is complicated by many factors such as cell retention, viability, structural and functional integration, and immune rejection. Therefore, in situ repair of the heart is best studied by targeting endogenous cardiac fibroblasts through viral transfection of the infarct zone. This approach was attempted in 2012 by Song et al. and Qian et al., in which local delivery of GM(H)T viruses induced reprogramming of nonmyocytes into iCMs by 4 weeks postsurgery [53, 54]. Additionally, Qian et al. reported that codelivery of thymosin *β*4 and GMT viruses further improved ejection fraction and reduced scar formation. Interestingly, it has been reported that the *in vivo* cardiac niche may improve the efficiency of reprogramming; however, the mechanisms underlying this observation remain elusive [27, 36, 53, 54]. Several studies have improved the *in vivo* reprogramming efficiency even further by optimizing polycistronic expression vectors to control the stoichiometry of TF expression or by the addition of small molecules delivered with TFs [27, 55–57].

## 6. Are All Fibroblasts Created Equal?

Cardiac fibroblasts are ideal targets for direct reprogramming as they are the most prominent cell type within the heart and play key roles in regulating normal myocardial function as well as adverse remodeling following injury. Various mouse and/or human fibroblast sources have been tested, including mouse embryonic fibroblasts, tail-tip fibroblasts, and dermal fibroblasts, with varying results, suggesting the importance of the starting cell type for direct reprogramming. It is also interesting to note that *in vivo* reprogramming has been reported to be more efficient than *in vitro* reprogramming, despite the fact that upon injury, cardiac fibroblast expresses TGF-*β*, which has been shown to be inhibitory to reprogramming *in vitro*. Furthermore, our lab has shown that cardiac fibroblasts are a heterogeneous population from different embryonic origins [58]. It is possible to postulate that perhaps a subpopulation of cardiac fibroblasts may be more susceptible to reprogramming depending on their developmental origin. Further understanding of the epigenetic landscape of fibroblasts and their susceptibility to direct reprogramming would be of great use to the field. This would also open up the possibility for repairing the heart by targeting specific fibroblast populations.

## 7. Nonintegrative Methods of Direct Reprogramming for Future *In vivo* Applications

The reprogramming results shown thus far suggest that direct reprogramming of fibroblasts can be a feasible therapeutic approach to repairing the injured myocardium. However, relatively safe methods for the delivery of various reprogramming factors need to be explored for *in vivo* applications. Adeno-associated virus (AAV) vectors are attractive tools for TF delivery, but the limited capacity of about 4.5 kb complicates the expression of multiple TFs in a single vector and still involves the use of an integrative viral system. Sendai virus reprogramming is an appealing alternative to AAV since it does not integrate into the host genome and has been successfully used to reprogram many different cell types to pluripotency; however, its use in direct reprogramming has not yet been explored. A recent study showed that acute expression of GMT in nonintegrating adenoviral vectors was as efficient as lentiviral vectors at reprogramming in a rat infarct model, which has increased the clinical applicability of *in vivo* reprogramming. As described in two recent reports, the temporal control and stoichiometric control of TFs are also important in determining reprogramming efficiency [57, 59]. Unfortunately, current *in vivo* viral reprogramming tools are unable to control dosage and temporal expression of TFs but warrant further investigation to improve reprogramming efficiency.

Compared to TFs and miRNAs, small molecules have many advantages such as more effective cell delivery and being nonimmunogenic and less expensive and are generally safer. Moreover, it is more convenient to control the process of reprogramming through varying small molecule concentrations and combinations *in vitro*. A combination of ascorbic acid, RepSox (a TGF-*β* inhibitor), forskolin, valproic acid, and CHIR99021 (a WNT pathway activator through the inhibition of glycogen synthase kinase 3) was shown to reprogram MEFs and mouse tail-tip fibroblasts to iCMs *in vitro* [60]. Cao et al. were able to use a cocktail of 9 small molecules (CHIR99021, A83-01, BIX01294, AS8351, SC1, OAC2, Y27632, SU16F, and JNJ10198409) to direct cardiac reprogramming of human foreskin fibroblasts *in vitro* before transplantation in injured murine hearts [61]. Among these small molecules were the epigenetic modifiers BIX01294 (a methyltransferase inhibitor) and AS8351 (a histone demethylase inhibitor), SC1 (an ERK2 and Ras-GAP inhibitor), OAC2 (an Oct4 activator), SU16F (a PDGFR*β* inhibitor), and JNJ10198409 (a PDGF receptor tyrosine kinase inhibitor). However, the use of small molecules for *in vivo* reprogramming poses some unanswered questions. Small molecules can enter the blood stream and spread to other organs with unknown consequences. Additionally, the ability of timely uptake into specific target cell type remains a challenge. Development of novel biomaterials for local delivery, controlled release, and retention of small molecules is still needed.

Another promising nonviral method of direct cardiac reprogramming is the use of modified mRNAs (modRNAs) [62]. ModRNAs are noncytopathic and do not integrate into the host genome, thus offering a safer approach to reprogramming. ModRNAs have been used successfully to generate induced pluripotent stem cells from somatic cells through transient expression of mRNAs that direct cell fate. ModRNAs are produced using an *in vitro* transcription system to generate mRNAs that contain a synthetic 5′ guanine cap and poly-A tail, which improves half-life and stability, as well as modified nucleotide bases that reduce the innate immune response of the host cell. This technology is endowed with a number of attractive properties that would make it a potentially powerful platform for direct cardiac reprogramming. ModRNAs can mediate robust and dose-titratable expression of key TFs over a specified time and in a particular sequence. Previous studies outlined in this review have highlighted the fact that direct cardiac reprogramming is a complex process that may require sequential treatments to better overcome the reprogramming barrier. ModRNAs may be ideal for direct reprogramming as they have a relatively short half-life; therefore, distinct factors can be expressed for a short period of time and then removed from the reprogramming cocktail or added again to continue expression. ModRNAs may open the door to following a more developmentally relevant sequence of TFs to improve transcription. It is also foreseeable that modRNAs could be combined with other small molecules, cytokines, and noncoding RNAs discussed in this review.

## 8. Roadblocks and Challenges

There has been significant progress in recent years with direct cardiac reprogramming through important discoveries in understanding the mechanism of reprogramming and the biology of cardiac development. However, several challenges must be addressed prior to clinical translation of this technology. The reprogramming efficiency must be increased in order to generate enough cells *in vitro* for transplantation. One avenue that has the potential to generate the number of cells needed for transplantation is reprogramming to CPCs, which can be expanded *in vitro* before transplantation. The retention, integration, and maturation of iCMs or CPCs after transplantation remain a concern. Multiplex immunostaining and patch clamp analysis have also revealed the presence of all three cardiomyocyte types (atrial, ventricular, and pacemaker) in iCMs, therefore increasing the risk of arrhythmias [16]. There is a need to develop techniques to generate specific subtypes of cardiomyocytes for both *in vitro* and *in vivo* direct reprogramming. A safe and effective approach to delivering and targeting reprogramming factors *in vivo* will be needed to circumvent *in vitro* reprogramming completely.

Transcription factors, inhibitors, cytokines, noncoding RNAs, and epigenetic modulators have been shown to be important for direct cardiac reprogramming. However, studies have uncovered variable reproducibility between different labs, leading to wide differences in reprogramming efficiency, maturity, and characteristics of the iCMs. These inconsistencies can be attributed to many factors other than the reprogramming factors themselves. First, the components of culture media used during reprogramming widely vary from group to group along with the duration of reprogramming before analysis. Additionally, the induction time, the type of fibroblasts, and the amount and sequence of factors used along with the time exposed to reprogramming factors are different between protocols. Moreover, the criteria used to measure the outcome and success are inconsistent and not standardized in the field. Reprogramming success is measured by some as the presence of cardiac-related structural proteins on immunostaining, while others employ a much more detailed approach including appearance of spontaneous beating along with gene and protein expression data. Even differences in the cardiac markers used to analyze the reprogramming efficiency, cardiac troponin T (cTnT) versus alpha myosin heavy chain (*α*MHC) versus GCaMP activity, and the method of measurement, flow cytometry versus immunofluorescence (IF), make comparisons among studies difficult. A myriad of criteria and stringency that have been used to evaluate reprogramming efficiency have been summarized by Addis and Epstein and are presented in Table 3 [63].

Optimization of the minimal yet sufficient combination of factors to improve reprogramming requires further research. Studies presented here have revealed that simply expressing a few core transcription factors is not sufficient for efficient cardiac reprogramming [64]. There may also be a dosage and temporal requirement for reprogramming factors [57, 59]. Other factors such as activated cellular signaling processes and epigenetic landscape should be considered to improve efficiency and quality. For example, when the TGF-*β* signaling pathway was disrupted by small molecules or when important cardiac regulatory miRNAs were added, an increase in reprogramming was observed, supporting the hypothesis that a multifaceted approach is likely necessary to achieve high reprogramming efficiency. Furthermore, these studies highlight the significant differences between mouse and human reprogramming as well as the effect of the starting fibroblast type (MEFs, tail-tip fibroblasts, or cardiac fibroblasts). Differences in fibroblast populations may be attributed to differences in epigenetic landscape, which can be influenced by many factors such as the cell environment or developmental origin. Further research on the heterogeneity of fibroblast epigenetic landscapes is warranted and will be of great benefit to direct reprogramming.

## 9. Conclusion

**Figure 2:**
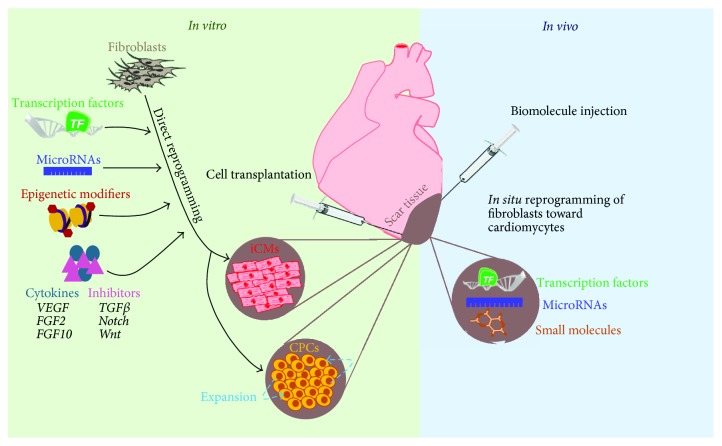
Schematic showing the current and future applications of direct cardiac reprogramming.

In this review, we discussed the reprogramming of fibroblasts into cardiomyocyte-like cells and expandable CPCs using transcription factors, small molecules, noncoding RNAs, and other biologics for the treatment of heart failure (Figure 2). Despite the current limitations that exist with direct cardiac reprogramming, this technology offers great promise for cardiac regeneration therapy. It is clear that the reprogramming process is very complex and that many factors have profound influence over this process. Continued research of key transcription factors, noncoding RNAs, small molecules, reprogramming mechanisms, delivery and targeting methods, and biomaterials will help advance direct cardiac reprogramming to large animal models and ultimately for the treatment of heart failure.

## Figures and Tables

**Table 1:** Summary of transcription factor direct cardiac reprogramming results.

Reprogramming factors	Mouse/human	*In vitro*/*in vivo*	Reported efficiency	Analysis method	Reference
GMT	Mouse	Both	4.8% cTnT+ *(in vitro*) 17% *α*MHC+ *(in vitro*)	FC	[11, 54]
GMHT	Mouse	Both	27.6% cTnT+ *(in vitro*)	FC	[16, 53]
GMHT, *MyoD* transactivation domain	Mouse	*In vitro*	19% cTnT+	IF	[17]
GMHT, *Nkx2–5*	Mouse	*In vitro*	1.6% GCaMP+	IF	[18]
MT, *Myocd*	Mouse	*In vitro*	12% cTnT+	FC	[19]
GMT, *Myocd*, *Srf*, *Mesp1*, *Smarcd3*	Mouse	*In vitro*	2.4% *α*MHC+	FC	[20]
*ETS2*, *MESP1*	Human	*In vitro*	13.7% *α*MHC+	FC	[21]
GMT, *MESP1*, *MYOCD*	Human	*In vitro*	5.9% cTnT+	FC	[22]
GMT, *ESSRG*, *MESP1*, *MYOCD*, *ZFPM2*	Human	*In vitro*	18.1% *α*MHC+	FC	[23]

FC: flow cytometry; IF: immunofluorescence.

**Table 2:** Summary of direct cardiac reprogramming results.

Reprogramming factors	Mouse/human	*In vitro*/*in vivo*	Reported efficiency	Analysis method	Reference
GMHT, SB431542	Mouse	*In vitro*	9.3% GCaMP+	IF	[26]
GMHT, DAPT	Mouse	*In vitro*	38% cTnT+	IF	[28]
GM(H)T, FGF2, FGF10, VEGF	Mouse	*In vitro*	2.9% cTnT+	FC	[32]
miRNA-1, miRNA-133, miRNA-208, miRNA-499	Mouse	*In vivo*	12% tdTomato+cTnT+	IHC	[36]
miRNA-1, miRNA-133, miRNA-208, miRNA-499, JAK inhibitor I	Mouse	Both	28% *α*MHC+ (*in vitro*)	FC	[30]
GMT, *Mesp1*, *Myocd*, miRNA-133	Mouse Human	*In vitro*	12.9% cTnT+ 27.8% cTnT+	FC	[35]
GHT, *MYOCD*, miRNA-1, miRNA-133	Human	*In vitro*	34.1% cTnT+	FC	[37]
GMHT, miRNA-1, miRNA-133, miRNA-208, miRNA-499, Y-27632, A83-01	Mouse	*In vitro*	60% cTnT+	IF	[29]
GMT, miRNA-590	Human Porcine	*In vitro*	4.6% cTnT+	FC	[39]
Ascorbic acid, RepSox, forskolin, valproic acid, CHIR99021	Mouse	*In vitro*	9% *α*MHC+	FC	[60]
CHIR99021, BIX01294, A83-01, AS8351, SC1, OAC2, Y27632, SU16F, JNJ10198409	Human	*In vitro*	6.6% cTnT+	FC	[61]

FC: flow cytometry; IF: immunofluorescence; IHC: immunohistochemistry.

**Table 3:** Criteria to evaluate reprogramming efficiency. Adapted from Addis and Epstein [63].

Characteristic	Stringency	Assay technique(s)
Gene expression	Low	RT-qPCR Reporter transgene
Protein expression	Low	Immunostaining Flow cytometry Western blot
Transcriptome and epigenetic analysis	High	Microarray RNA-seq ChIP-seq ATAC-seq
Contraction and force generation	High	Spontaneous Chemical stimulation Electrical stimulation Three-dimensional bioengineered platforms
Electrophysiological	High	Patch clamp Microelectrode arrays Optical mapping
Calcium transients and electrical coupling	High	Calcium-sensitive dyes Genetically encoded indicators (GCaMP) Optical mapping
Functional improvement	High	Echocardiography

## References

[1] Braunwald E. (2015). The war against heart failure: the *Lancet* lecture. *The Lancet*.

[2] Heidenreich P. A., Albert N. M., Allen L. A. (2013). American Heart Association Advocacy Coordinating Committee, Council on Arteriosclerosis, Thrombosis and Vascular Biology, Council on Cardiovascular Radiology and Intervention, Council on Clinical Cardiology, Council on Epidemiology and Prevention, Stroke Council. Forecasting the impact of heart failure in the United States: a policy statement from the American Heart Association. *Circulation: Heart Failure*.

[3] Cahill T. J., Choudhury R. P., Riley P. R. (2017). Heart regeneration and repair after myocardial infarction: translational opportunities for novel therapeutics. *Nature Reviews Drug Discovery*.

[4] Taylor C. J., Roalfe A. K., Iles R., Hobbs F. D. (2012). Ten-year prognosis of heart failure in the community: follow-up data from the Echocardiographic Heart of England Screening (ECHOES) study. *European Journal of Heart Failure*.

[5] Davis R. L., Weintraub H., Lassar A. B. (1987). Expression of a single transfected cDNA converts fibroblasts to myoblasts. *Cell*.

[6] Choi J., Costa M. L., Mermelstein C. S., Chagas C., Holtzer S., Holtzer H. (1990). MyoD converts primary dermal fibroblasts, chondroblasts, smooth muscle, and retinal pigmented epithelial cells into striated mononucleated myoblasts and multinucleated myotubes. *Proceedings of the National Academy of Sciences of the United States of America*.

[7] Vierbuchen T., Ostermeier A., Pang Z. P., Kokubu Y., Südhof T. C., Wernig M. (2010). Direct conversion of fibroblasts to functional neurons by defined factors. *Nature*.

[8] Yu B., He Z. Y., You P. (2013). Reprogramming fibroblasts into bipotential hepatic stem cells by defined factors. *Cell Stem Cell*.

[9] Huang P., He Z., Ji S. (2011). Induction of functional hepatocyte-like cells from mouse fibroblasts by defined factors. *Nature*.

[10] Xie H., Ye M., Feng R., Graf T. (2004). Stepwise reprogramming of B cells into macrophages. *Cell*.

[11] Ieda M., Fu J. D., Delgado-Olguin P. (2010). Direct reprogramming of fibroblasts into functional cardiomyocytes by defined factors. *Cell*.

[12] Hiroi Y., Kudoh S., Monzen K. (2001). Tbx5 associates with Nkx2-5 and synergistically promotes cardiomyocyte differentiation. *Nature Genetics*.

[13] Perrino C., Rockman H. A. (2006). GATA4 and the two sides of gene expression reprogramming. *Circulation Research*.

[14] Dodou E., Verzi M. P., Anderson J. P., Xu S. M., Black B. L. (2004). *Mef2c* is a direct transcriptional target of ISL1 and GATA factors in the anterior heart field during mouse embryonic development. *Development*.

[15] Dai Y. S., Cserjesi P., Markham B. E., Molkentin J. D. (2002). The transcription factors GATA4 and dHAND physically interact to synergistically activate cardiac gene expression through a p300-dependent mechanism. *Journal of Biological Chemistry*.

[16] Nam Y. J., Lubczyk C., Bhakta M. (2014). Induction of diverse cardiac cell types by reprogramming fibroblasts with cardiac transcription factors. *Development*.

[17] Hirai H., Katoku-Kikyo N., Keirstead S. A., Kikyo N. (2013). Accelerated direct reprogramming of fibroblasts into cardiomyocyte-like cells with the MyoD transactivation domain. *Cardiovascular Research*.

[18] Addis R. C., Ifkovits J. L., Pinto F. (2013). Optimization of direct fibroblast reprogramming to cardiomyocytes using calcium activity as a functional measure of success. *Journal of Molecular and Cellular Cardiology*.

[19] Protze S., Khattak S., Poulet C., Lindemann D., Tanaka E. M., Ravens U. (2012). A new approach to transcription factor screening for reprogramming of fibroblasts to cardiomyocyte-like cells. *Journal of Molecular and Cellular Cardiology*.

[20] Christoforou N., Chellappan M., Adler A. F. (2013). Transcription factors MYOCD, SRF, Mesp1 and SMARCD3 enhance the cardio-inducing effect of GATA4, TBX5, and MEF2C during direct cellular reprogramming. *PLoS One*.

[21] Islas J. F., Liu Y., Weng K. C. (2012). Transcription factors ETS2 and MESP1 transdifferentiate human dermal fibroblasts into cardiac progenitors. *Proceedings of the National Academy of Sciences of the United States of America*.

[22] Wada R., Muraoka N., Inagawa K. (2013). Induction of human cardiomyocyte-like cells from fibroblasts by defined factors. *Proceedings of the National Academy of Sciences of the United States of America*.

[23] Fu J. D., Stone N. R., Liu L. (2013). Direct reprogramming of human fibroblasts toward a cardiomyocyte-like state. *Stem Cell Reports*.

[24] Ao A., Hao J., Hopkins C. R., Hong C. C. (2012). DMH1, a novel BMP small molecule inhibitor, increases cardiomyocyte progenitors and promotes cardiac differentiation in mouse embryonic stem cells. *PLoS One*.

[25] Cai W., Guzzo R. M., Wei K., Willems E., Davidovics H., Mercola M. (2012). A nodal-to-TGF*β* cascade exerts biphasic control over cardiopoiesis. *Circulation Research*.

[26] Ifkovits J. L., Addis R. C., Epstein J. A., Gearhart J. D. (2014). Inhibition of TGF*β* signaling increases direct conversion of fibroblasts to induced cardiomyocytes. *PLoS One*.

[27] Mohamed T. M., Stone N. R., Berry E. C. (2017). Chemical enhancement of in vitro and in vivo direct cardiac reprogramming. *Circulation*.

[28] Abad M., Hashimoto H., Zhou H. (2017). Notch inhibition enhances cardiac reprogramming by increasing MEF2C transcriptional activity. *Stem Cell Reports*.

[29] Zhao Y., Londono P., Cao Y. (2015). High-efficiency reprogramming of fibroblasts into cardiomyocytes requires suppression of pro-fibrotic signalling. *Nature Communications*.

[30] Jayawardena T. M., Egemnazarov B., Finch E. A. (2012). MicroRNA-mediated in vitro and in vivo direct reprogramming of cardiac fibroblasts to cardiomyocytes. *Circulation Research*.

[31] Zhou H., Dickson M. E., Kim M. S., Bassel-Duby R., Olson E. N. (2015). Akt1/protein kinase B enhances transcriptional reprogramming of fibroblasts to functional cardiomyocytes. *Proceedings of the National Academy of Sciences of the United States of America*.

[32] Yamakawa H., Muraoka N., Miyamoto K. (2015). Fibroblast growth factors and vascular endothelial growth factor promote cardiac reprogramming under defined conditions. *Stem Cell Reports*.

[33] Rao P. K., Toyama Y., Chiang H. R. (2009). Loss of cardiac microRNA-mediated regulation leads to dilated cardiomyopathy and heart failure. *Circulation Research*.

[34] Liu N., Bezprozvannaya S., Williams A. H. (2008). MicroRNA-133a regulates cardiomyocyte proliferation and suppresses smooth muscle gene expression in the heart. *Genes & Development*.

[35] Muraoka N., Yamakawa H., Miyamoto K. (2014). MiR-133 promotes cardiac reprogramming by directly repressing Snai1 and silencing fibroblast signatures. *The EMBO Journal*.

[36] Jayawardena T. M., Finch E. A., Zhang L. (2015). MicroRNA induced cardiac reprogramming in vivo: evidence for mature cardiac myocytes and improved cardiac function. *Circulation Research*.

[37] Nam Y. J., Song K., Luo X. (2013). Reprogramming of human fibroblasts toward a cardiac fate. *Proceedings of the National Academy of Sciences of the United States of America*.

[38] Eulalio A., Mano M., Ferro M. D. (2012). Functional screening identifies miRNAs inducing cardiac regeneration. *Nature*.

[39] Singh V. P., Mathison M., Patel V. (2016). MiR-590 promotes transdifferentiation of porcine and human fibroblasts toward a cardiomyocyte-like fate by directly repressing specificity protein 1. *Journal of the American Heart Association*.

[40] Johnsson P., Lipovich L., Grandér D., Morris K. V. (2014). Evolutionary conservation of long non-coding RNAs; sequence, structure, function. *Biochimica et Biophysica Acta (BBA) - General Subjects*.

[41] Gomes C. P. C., Spencer H., Ford K. L. (2017). The function and therapeutic potential of long non-coding RNAs in cardiovascular development and disease. *Molecular Theraphy Nucleic Acids*.

[42] Klattenhoff C. A., Scheuermann J. C., Surface L. E. (2013). *Braveheart*, a long noncoding RNA required for cardiovascular lineage commitment. *Cell*.

[43] Xue Z., Hennelly S., Doyle B. (2016). A G-rich motif in the lncRNA *Braveheart* interacts with a zinc-finger transcription factor to specify the cardiovascular lineage. *Molecular Cell*.

[44] Grote P., Wittler L., Hendrix D. (2013). The tissue-specific lncRNA *Fendrr* is an essential regulator of heart and body wall development in the mouse. *Developmental Cell*.

[45] Wang Z., Zhang X. J., Ji Y. X. (2016). The long noncoding RNA *Chaer* defines an epigenetic checkpoint in cardiac hypertrophy. *Nature Medicine*.

[46] Wang Z., Wang Y. (2015). Dawn of the epi-LncRNAs: new path from Myheart. *Circulation Research*.

[47] Liu Z., Chen O., Zheng M. (2016). Re-patterning of H3K27me3, H3K4me3 and DNA methylation during fibroblast conversion into induced cardiomyocytes. *Stem Cell Research*.

[48] Zhou Y., Wang L., Vaseghi H. R. (2016). Bmi1 is a key epigenetic barrier to direct cardiac reprogramming. *Cell Stem Cell*.

[49] Liu Z., Wang L., Welch J. D. (2017). Single-cell transcriptomics reconstructs fate conversion from fibroblast to cardiomyocyte. *Nature*.

[50] Lalit P. A., Salick M. R., Nelson D. O. (2016). Lineage reprogramming of fibroblasts into proliferative induced cardiac progenitor cells by defined factors. *Cell Stem Cell*.

[51] Lalit P. A., Rodriguez A. M., Downs K. M., Kamp T. J. (2017). Generation of multipotent induced cardiac progenitor cells from mouse fibroblasts and potency testing in ex vivo mouse embryos. *Nature Protocols*.

[52] Zhang Y., Cao N., Huang Y. (2016). Expandable cardiovascular progenitor cells reprogrammed from fibroblasts. *Cell Stem Cell*.

[53] Song K., Nam Y. J., Luo X. (2012). Heart repair by reprogramming non-myocytes with cardiac transcription factors. *Nature*.

[54] Qian L., Huang Y., Spencer C. I. (2012). *In vivo* reprogramming of murine cardiac fibroblasts into induced cardiomyocytes. *Nature*.

[55] Ma H., Wang L., Yin C., Liu J., Qian L. (2015). *In vivo* cardiac reprogramming using an optimal single polycistronic construct. *Cardiovascular Research*.

[56] Mathison M., Singh V. P., Gersch R. P. (2014). “Triplet” polycistronic vectors encoding Gata4, Mef2c, and Tbx5 enhances postinfarct ventricular functional improvement compared with singlet vectors. *The Journal of Thoracic and Cardiovascular Surgery*.

[57] Wang L., Liu Z., Yin C. (2015). Stoichiometry of Gata4, Mef2c, and Tbx5 influences the efficiency and quality of induced cardiac myocyte reprogramming. *Circulation Research*.

[58] Ali S. R., Ranjbarvaziri S., Talkhabi M. (2014). Developmental heterogeneity of cardiac fibroblasts does not predict pathological proliferation and activation. *Circulation Research*.

[59] Umei T. C., Yamakawa H., Muraoka N. (2017). Single-construct polycistronic doxycycline-inducible vectors improve direct cardiac reprogramming and can be used to identify the critical timing of transgene expression. *International Journal of Molecular Sciences*.

[60] Fu Y., Huang C., Xu X. (2015). Direct reprogramming of mouse fibroblasts into cardiomyocytes with chemical cocktails. *Cell Research*.

[61] Cao N., Huang Y., Zheng J. (2016). Conversion of human fibroblasts into functional cardiomyocytes by small molecules. *Science*.

[62] Warren L., Manos P. D., Ahfeldt T. (2010). Highly efficient reprogramming to pluripotency and directed differentiation of human cells with synthetic modified mRNA. *Cell Stem Cell*.

[63] Addis R. C., Epstein J. A. (2013). Induced regeneration--the progress and promise of direct reprogramming for heart repair. *Nature Medicine*.

[64] Chen J. X., Krane M., Deutsch M. A. (2012). Inefficient reprogramming of fibroblasts into cardiomyocytes using Gata4, Mef2c, and Tbx5. *Circulation Research*.

